# The impact of immersive video learning on speech-language pathology students’ dysphagia education: a mixed-methods study

**DOI:** 10.1186/s12909-026-08630-z

**Published:** 2026-01-27

**Authors:** Raymond Fong, Wilson S. Yu, Connie C. Y. Kwan

**Affiliations:** 1 Department of Otorhinolaryngology, head and neck surgery, Faculty of Medicine, The Chinese University of Hong Kong, Hong Kong SAR, China; 2 Institute of Human Communicative Research, The Chinese University of Hong Kong, Hong Kong SAR, China

**Keywords:** Dysphagia, Speech language pathology, Immersive, Virtual reality

## Abstract

**Background:**

Dysphagia education for speech-language pathology (SLP) students is challenged by limited hands-on exposure and skill transfer to diverse clinical settings such as hospital, aged-care facilities and clinics. Video-based learning, including immersive technologies, offers a potential solution for standardized, safe exposure.

**Methods:**

This mixed-methods study randomized 65 first-year SLP Master’s students into immersive (*n* = 33) and non-immersive (*n* = 32) video groups. Immersive videos were delivered via Meta Quest 3 headsets, non-immersive as MP4 links. Learning outcomes were assessed through clinical placement evaluations. Student perceptions were collected using Likert scales and open-ended questions.

**Results:**

The immersive group achieved significantly higher clinical placement performance (*p* = 0.030), especially in assessment planning, oromotor/ swallow trial procedures, and clinical reasoning. Immersive videos provided a greater sense of presence (*p* = 0.020). However, non-immersive videos were viewed longer (142.27 vs. 47.63 min, *p* = 0.002) and perceived as easier to navigate (*p* = 0.001) and less overwhelming (*p* = 0.002). Qualitative data highlighted immersive group challenges (physical discomfort, logistical barriers, usability) and non-immersive group challenges (content quality, camera angles).

**Conclusions:**

Immersive videos enhance practical clinical skills and presence in dysphagia education but face usability and logistical hurdles. Non-immersive videos offer high accessibility and user satisfaction, promoting sustained engagement. A blended learning approach, leveraging immersive videos for high-stakes procedural training and non-immersive for conceptual understanding and review, is recommended to optimize SLP dysphagia education, balancing pedagogical effectiveness with practical usability.

**Supplementary Information:**

The online version contains supplementary material available at 10.1186/s12909-026-08630-z.

## Background

### Dysphagia education

Dysphagia, or swallowing difficulty, is a complex condition that significantly impacts patient health and quality of life [1]. For speech language pathology (SLP) students, acquiring comprehensive knowledge and practical skills in dysphagia assessment and intervention is paramount to providing competent and safe patient care [2]. Dysphagia management requires clinical reasoning, execution of procedural tasks, and observation of responses by the patient and carer [2]. These have to be conducted while maintaining professional interaction with the patient and caregiver, and often in a busy clinical environment. In most dysphagia training curricula, theoretical knowledge are taught first followed by clinical practicum in settings where SLPs practice [3]. They include hospitals, aged-care facilities and clinics.

Unlike medical or nursing training, which is predominantly centered in hospital environments, SLP practica span a vast array of settings, including acute hospitals, schools, and community-based aged-care facilities. This diversity makes early and repeated exposure to every potential clinical setting impractical during the foundational years of training. Consequently, students often struggle with the transference of clinical skills, particularly in complex areas like dysphagia [4, 5]. This lack of environmental familiarity leads to limited opportunities for direct, hands-on patient exposure prior to clinical practicum. Ultimately, these limitations restrict the depth and breadth of practical learning experiences necessary for developing clinical proficiency before students enter high-stakes clinical environments.

To address these challenges, simulations in physical environments that mimic real-world clinical scenarios have been adopted in dysphagia education [6–8]. It has shown to be beneficial for students but it cannot fully replace the experience of stepping into the clinical field [7]. The unfamiliarity with these environments may result in increased anxiety for students [5]. If students can be more prepared for these environments, the learning efficiency and outcomes could be enhanced.

### Role of video-based learning

In response to these challenges, video-based learning modalities have emerged as a viable and increasingly adopted solution in health professions education [9, 10]. These tools offer standardized, repeatable, and accessible exposure to a diverse range of clinical scenarios without compromising patient safety and ethical concerns [11]. Non-immersive videos of simulated or real patient clinical encounters, typically delivered via online links or institutional learning management systems, have been widely utilized due to their ease of access and flexibility, allowing students to review content at their own pace and convenience [9].

Video-based learning has been demonstrated to improve engagement, accessibility and flexibility in learning [11]. A meta-analysis also showed that a significant effect on knowledge acquisition was demonstrated through video-based learning in dentistry and medicine, but not in nursing [11]. This highlighted the inherent differences in various fields of medical education might not have the same outcome despite using the same modality. It cannot be assumed that the effect of video-based learning can be applicable to all fields of medical education.

## Emergence of immersive technologies

Beyond traditional video formats, the field of medical education has witnessed the emergence of immersive learning technologies, particularly immersive videos with or without virtual reality (VR) or augmented reality (AR) components [12]. Immersive videos are designed to allow viewers to have a better immersion in real-world scenarios, offering a heightened “sense of presence” and “realism” for the learner [9]. By the theory of Cone of Experience [13], more concrete instructional resources and experiences were suggested to lead to better outcomes. Immersive videos, as compared to still or motion pictures, are more concrete. It also appeals to a broad range of learners and was deemed a versatile tool for medical education [9]. This technology holds promise for providing highly engaging and experiential learning opportunities that more closely mimic actual clinical encounters, viewers of the immersive videos can appreciate all the events that happen during the clinical encounter, while immersed in the environment [14]. The viewer would have more of a first-person presence in those videos compared with non-immersive videos.

In a scoping review published in 2021 [9], 14 studies that investigated immersive video usage in health education were included for analysis. The review found that immersive videos resulted in improved attention, and associated with skill enhancement and improvement in usability and user satisfaction [9]. In another review in 2022 [10], 29 randomized controlled studies on immersive videos against traditional learning modalities were included. Most of the studies were conducted on medical students and nursing students. The study concluded that knowledge gain was found to be equal between the two but the learning experience increased with immersive technologies [10]. Both reviews were positive about immersive technology in terms of learning gains and outcomes [9, 10] and concluded that it could be a viable alternative in some aspects in health education.

### Research gap and rationale

Despite the growing interest and application of immersive video technologies in medical education, there remains a notable gap in the literature regarding direct comparative studies, especially within specialized areas such as dysphagia education for SLP students. There has been only one related study that investigated the use of mixed reality simulator on interprofessional communication in educational SLP education [15]. In this study, 80 students were randomized into 4 groups with two different variables: mixed reality versus traditional role play, and with coaching versus without coaching. The study did not find mixed reality to be significantly more superior than role play, the key factor was the presence of coaching [15]. However, the main technology applied in this study was not immersive videos and it was also not in the dysphagia education domain.

Moreover, existing research often focuses on the efficacy of one modality in isolation or lacks a comprehensive assessment that integrates both objective learning outcomes (e.g., clinical skills, knowledge acquisition) and subjective user experience [9]. This study aims to bridge this gap by systematically comparing the impact of immersive versus non-immersive videos, thereby generating empirical evidence to inform and guide curriculum development, pedagogical strategies, and the integration of technology within SLP education programs.

### Study objectives

The primary objectives of this study are twofold: first, to systematically compare the impact of immersive versus non-immersive videos on SLP students’ dysphagia education outcomes, specifically assessing early performance on clinical placement evaluations; and second, to evaluate student perceptions of the respective learning experiences between the two modalities. It was hypothesized that immersive videos might lead to a higher sense of engagement and presence, potentially translating into superior practical skill transfer, while non-immersive videos might offer greater ease of access and overall user satisfaction.

## Methodology

The research was approved by the Survey and Behavioural Research Ethics Committee of the Chinese University of Hong Kong (Ref. no.: SBRE-24–0511). This investigation employed a mixed-methods approach, combining a quantitative comparative study with a qualitative component. The study utilized a two-group experimental design to compare the learning effectiveness and student perceptions associated with immersive and non-immersive video learning modalities.

### Participants and recruitment

Participants were recruited through a convenience sampling method from a specific SLP training program, with voluntary participation ensuring ethical compliance. Participants were first year SLP students in a 2-year Masters level program. Students were in their second and third semester during data collection. In the second semester, they had a 39-hour face-to-face taught course on dysphagia. In the third semester following, each student were assigned randomly to their first clinical practicums on adult patients in various settings such as aged-care facilities and community outpatient settings. The number of students in the class at the start of the study was 65. All 65 students in the course provided informed written consent to participate in the study.

### Intervention

Students were already divided into 12 groups for teaching and learning purposes at the start of the semester, each group had 5–6 students. For this study, 6 groups (33 students) were randomly assigned as immersive group and 6 groups (32 students) as non-immersive groups. All students had access to videos for 6 weeks from 1 Mar 2025 to 15 Apr 2025. The videos were produced using a Insta360 × 4 camera (Arashi Vision Inc., Shenzhen, China), while the first author was performing swallowing assessment for 4 patients thus it can be categorized as a demonstration video [16]. The content and length of the videos are detailed in Table 1.

**Table 1:** Content and length of the immersive and non-immersive videos

Patient	Clinical information	Part	Length
Patient A	*Wheelchair*	Case history taking from patient and carer	11:49
	*Stroke*,* on tube feeding*	Communication screening	7:45
	*Hospital ward*	Cranial nerve and oromotor assessment	5:18
		Motor speech assessment	3:10
		Pre-swallow tasks	3:56
		Swallow trials	4:59
		Summary of session to patient and carer	5:22
		Total duration	42:19
Patient B	*Wheelchair*	Oromotor and cranial nerve examination	6:03
	*Stroke*,* on tube feeding*	Swallow trials	13:26
	*Outpatient clinic*		
		Total duration	19:29
Patient C	*Ambulatory*	Oromotor and cranial nerve assessment	3:00
	*Dementia*	Swallow trials	2:50
	*Nursing Home*		
		Total duration	5:50
Patient D	*Ambulatory*	Oromotor and cranial nerve assessment	3:40
	*Pseudobulbar palsy*	Motor speech assessment	3:04
	*Outpatient clinic*	Swallow trials	5:42
		Total duration	12:26

Immersive videos were produced by exporting the trimmed raw videos, no further editing was conducted. Non-immersive videos were produced by automatically tracking either the clinician or patient in the raw videos with the software Insta360 Studio (Version 5.2.4). The length and content of the immersive and non-immersive videos were the same. The medical information of each patient and the clinical reasoning applied by the clinician during the assessment were provided to all students as a printed document. All students were provided with the instructions that the videos would not be available to them after 6 weeks and were encouraged to use them as learning material for the dysphagia course.

### Immersive group

Students in the immersive groups were provided with 360-degree immersive videos that were preloaded onto a Meta Quest 3 headset (Meta Inc., California, USA), along with written instructions on the operation of the headset. The use of a dedicated VR headset aimed to provide a high-fidelity, interactive learning environment and ensured adherence to recommendation [16]. Each group of 5–6 students shared 1 headset due to resource constraints. Using the same headset ensured that they would not have different learning experience because of the difference in hardware. The exact duration each student had the headset for was not documented and would vary among students, but they were encouraged to share it among themselves evenly during the 6-week period.

### Non-immersive group

Students in the non-immersive group were provided with videos in.mp4 format through links that could only be accessed and viewed with their individual account on Microsoft OneDrive (Microsoft Inc., Seattle, USA). Having the files accessible through links allowed for flexible viewing through standard computing devices. They were advised not to share the videos with any other students throughout the 6-week period.

### Outcome measures

#### Learning outcomes (quantitative)

Objective learning outcomes were measured through three distinct assessments. First, scores were collected from three Objective Structured Clinical Examination (OSCE) questions that were designed to assess pre-clinical skills in dysphagia assessment and intervention. The OSCE examination occurred 1 week after the end of access of video and prior to clinical practicum. Three questions out of 12 in the OSCE were analyzed for this study, the questions examined students’ competence on thickened liquid preparation, providing instructions for swallowing maneuvers and executing a part of the cranial nerve examination. The other OSCE questions were not included in analysis as they were unrelated to dysphagia assessment and intervention. Secondly, scores were obtained from the final written examination of the course, which occurred 2 days after the end of access of the videos. The written exam had two sections each carrying 50% of the marks: 20 multiple choice questions and two case-based essay-style questions. The total score from the exam was used for analysis. Thirdly, the evaluation of students’ clinical placement performance by practicum supervisors was obtained through an online questionnaire that was sent to all clinical supervisors (*n* = 23). The clinical supervisor to student ratio ranged from 1:2 to 1:4. The supervisors were blinded to which group each student was assigned to. The questionnaire (Supplementary material 1) was sent 3 weeks after commencement of the 10-week clinical practicum. They were given 1 week to respond. Information regarding the years of experience, placement site setting and the number of sessions completed were collected. For each student, the supervisor was asked to rate 9 areas using a Likert Scale (1: Far below expectation; 2: Below Average; 3: Average; 4: Above Average; 5: Far beyond expectation). The mean score was calculated as the clinical placement performance metric. This metric served as the primary quantitative measure for applied clinical skills and professional competence.

#### Perception and views (quantitative & qualitative)

Student perceptions and views were gathered using a comprehensive questionnaire (Supplementary material 1) 1 week after the video availability period. All participants were invited to respond anonymously within 1 week, which was prior to the commencement of the clinical placement to avoid confounding of experience.

### Data analysis

#### Quantitative analysis

Descriptive statistics, including means and standard deviations or median and interquartile range, were calculated and presented for all quantitative outcome measures for both the immersive and non-immersive groups. Inferential statistical tests, such as independent t-tests or Mann-Whitney U tests, were employed to compare the two groups, depending on the distribution of the data and sample size. Missing data were handled using listwise deletion for complete cases to ensure valid comparisons.

#### Qualitative analysis

The open-ended responses from the perception questionnaire were subjected to thematic analysis [17]. The analysis followed six distinct phases: familiarization with the data, generating initial codes, searching for themes, reviewing themes, defining and naming themes, and producing the final report. To ensure trustworthiness, two investigators (RF and CK) independently coded the transcripts. Following independent coding, consensus meetings were held to resolve any discrepancies in code application and to refine the thematic structure.

## Results

### Participant demographics and performance metrics

The study initially had 65 participants, with 61 female and 4 male. The mean age of the participants were 27.64 years (SD = 5.05 years), range 22 to 42 years. The pre-clinical placement performance of 65 participants in OSCE and written exam were analyzed by independent t-test and they were not significantly different between the two groups (Table 2). For the clinical practicum data, only 18 out of 23 (78.3%) supervisors responded by the 1-week deadline, which accounted for 49 (75.4%) students. The years of experience of supervisor and number of sessions did not differ between the groups (Table 2). For the practicum settings, the distribution were similar. For the immersive video group, 20 students were assigned to nursing home and 13 were assigned to community outpatient settings while for the non-immersive group, 22 were assigned to nursing home and 9 were assigned to community outpatient settings.

**Table 2:** Mean and standard deviation and comparison by independent t-test of pre-clinical performance and clinical practicum variables by group

Pre-clinical placement performance	Immersive Group (*n* = 33)	Non-Immersive Group (*n* = 32)	t	*p*
OSCE Total score	27.12 ± 2.41	26.37 ± 3.34	1.036	0.304
Written exam total score	47.48 ± 11.18	45.03 ± 10.82	0.898	0.372
**Clinical practicum variables**	**Immersive Group** **(*n* = 25)**	**Non-Immersive Group (** ***n*** ** = 24)**		
Years of experience of supervisor	3.28 ± 3.77	3.35 ± 5.16	0.023	0.982
Number of sessions	3.16 ± 0.75	3.25 ± 1.42	−0.279	0.781

For the primary outcome of the study, the immersive group demonstrated a significantly higher score for clinical placement performance by Mann-Whitney U test, *U* = 192.0, *z* = −2.17, *p* = 0.030, as measured by the composite outcome metric (Table 3). This metric, representing the evaluation of applied clinical skills by supervisors, suggests a unique and potentially substantial strength of immersive learning in fostering practical, applied clinical skills. For the sub-items, participants in the immersive group were significantly better for assessment planning, clinical procedures of oromotor examination and swallow trials and also clinical reasoning (Table 3). For the other 5 sub-items, no significant difference were found between the two groups (Table 3).

**Table 3:** Median and interquartile range and comparison by Mann-Whitney U test of clinical practicum performance between groups

	Immersive Group (*n* = 25)	Non-Immersive Group (*n* = 24)	U	Z	*p*
**Composite outcome metric**	3 (0.67)	3(0.67)	192.0	−2.17	0.030*
Assessment planning	3 (0)	3 (1)	196.0	−2.35	0.019*
Familiarity with environment	3 (0)	3 (1)	231.5	−1.62	0.105
Clinical procedure – Communication assessment	3 (0)	3 (2)	241.5	−1.32	0.187
Clinical procedure – Oromotor and cranial nerve assessment	3 (0)	2 (1)	178.5	−2.70	0.007*
Clinical procedure – Swallow trials	3 (0)	2 (2)	146.0	−3.33	0.001*
Handling of clinical material	3 (0)	3 (1)	252.0	−1.08	0.281
Clinical reasoning	3 (1)	2 (1)	157.0	−3.16	0.002*
Interaction with patient/carer	3 (1)	3 (0)	256.5	−0.96	0.335
Time management	3 (0)	3 (1)	214.5	−1.86	0.063

*Indicate *p* < 0.05

### Student perceptions (quantitative findings)

For the questionnaire on students’ perception, out of the 65 participants, 49 (75.4%) unique and complete responses were collected by the 1-week period. For the time spent on viewing the videos, an independent t-test was run to determine if there were differences between the two groups. The non-immersive group reported longer viewing time (*M* = 142.27 min, *SD* = 148.55) than immersive group (*M* = 47.63 min, *SD* = 32.63), a statistically significant difference, *M* = −94.64, 95%CI [−153.7, −35.58], t(47) = −3.224, *p* = 0.002. Table 4 summarizes the descriptive statistics and statistical comparisons by Mann-Whitney U test for all Likert scale perception questions. Among the perception measures, students found that immersive videos provided a greater sense of presence and immersion. However, they were significantly more likely to recommend non-immersive video and found non-immersive videos significantly easier to navigate. The technology was significantly more overwhelming for immersive video and took significantly longer to get accustomed to it.

**Table 4:** Median and interquartile range and comparison by Mann-Whitney U test of students’ perceptions (Likert Scale) between groups

Perception Measure	Immersive (*n* = 27)	Non-immersive (*n* = 22)	U	Z	*p*
Engagement and realism					
Actively involved	4.0 (1.0)	3.5 (1.0)	225.5	−1.55	0.121
Engaging	4.0 (0)	4.0 (1.0)	284.5	−0.29	0.773
Held attention	4.0 (0)	4.0 (1.25)	278.0	−0.42	0.678
Sense of presence/Immersion	4.0 (1.0)	3.0 (2.0)	186.5	−2.33	0.020*
Realism of scenarios	4.0 (2.0)	4.0 (2.0)	277.5	−0.42	0.677
Usefulness					
Familiarity with clinical setting	4.0 (2.0)	4.0 (1.0)	280.5	−0.36	0.722
Familiarity with clinical procedures	4.0 (1.0)	4.0 (1.0)	254.5	−0.99	0.321
Understanding of clinical reasoning	4.0 (1.0)	4.0 (0)	250.0	−1.12	0.263
Met learning expectations	4.0 (1.0)	4.0 (0)	235.0	−1.50	0.133
Likelihood to recommend to peers	4.0 (1.0)	4.0 (1.0)	190.0	−2.36	0.018*
Technical aspects					
Ease of navigation	3.0 (1.0)	5.0 (1.0)	138.0	−3.35	0.001*
Instructions clarity	4.0 (1.0)	4.0 (0.25)	237.5	−1.36	0.175
Ease of access to material	4.0 (2.0)	4.5 (1.0)	177.5	−2.54	0.011*
Accessibility for diverse needs	4.0 (1.0)	4.0 (1.0)	263.0	−0.74	0.458
Technology too overwhelming	3.0 (2.0)	1.5 (2.0)	148.5	−3.09	0.002*
Time needed to get accustomed	4.0 (2.0)	1.5 (2.0)	133.5	−3.40	0.001*
Overall Satisfaction	4.0 (1.0)	4.0 (0)	245.0	−1.20	0.231

### Student perceptions (qualitative findings)

Qualitative feedback from the immersive group highlighted several specific challenges. These included significant physical discomfort, with students frequently reporting the headset as “heavy,” causing “dizziness,” “neck pain,” or being “tiring for both my eyes and head.” Logistical barriers were also prominent, such as difficulties related to device sharing within groups (“only have 1 headset and we need to circulate”), limited access time (“cant access the videos anytime I need to refresh my memory”), and concerns about “easy to run out of battery.” Usability deficiencies were noted, with students unfamiliar with the VR tool, experiencing navigation issues (“hard to navigate at the beginning”), and the inability to “jot notes while watching the videos with the headset on”. Visual clarity issues were also raised, including “the angle of camera makes it difficult to see the patient’s movements”, “vision was blocked”, “vision is a little bit distorted at the edges”, and the impact of pre-existing visual impairments.

For the non-immersive group, challenges primarily revolved around content quality, such as “voices were not very clear” and “the video appears to be so blurred”, indicating basic production quality concerns. Pedagogical gaps were also identified, with students expressing a desire for enhanced learning support, including “subtitles”, “annotations to assist the learning”, explanations of “clinical judgement” and “a summary of the results.” Similar to the immersive group, concerns were raised about suboptimal camera angles, such as “the clinician accidentally standing in front of the camera” or the “camera was put a bit far away”.

Across both modalities, common improvement themes emerged from the qualitative data. These included calls for improved video quality (resolution, audio), optimized camera angles (closer views, clearer perspectives, potentially first-person views), and enhanced pedagogical support (subtitles, annotations, explicit clinical reasoning explanations, and summary notes).

## Discussion

### Key findings

This study investigated the comparative impact of immersive versus non-immersive video learning on Speech-Language Pathology students’ dysphagia education. The quantitative findings indicate students who had access to the immersive videos showed a significantly higher mean score in clinical placement evaluations, although causality cannot be fully established due to potential confounders. Qualitatively, immersive videos delivered a superior mean sense of presence and realism, but this was counterbalanced by significant usability and logistical challenges, leading to lower mean overall satisfaction and recommendation rates compared to non-immersive videos. Non-immersive videos, conversely, were perceived as highly accessible, user-friendly for ease of navigation and without overwhelming technology. Non-immersive videos also fostered more sustained engagement, as evidenced by significantly longer viewing times.

### Interpretation of learning outcome differences

Through between group comparisons, students’ performance in written exam and OSCE examinations were not significantly different. The placement variables such as number of sessions and years of experience of supervisors did not differ. This indicated that the two groups (immersive and non-immersive) were largely similar in terms of their academic performance and placement nature. The observed performance differences in placement between the group, with the students who had received immersive video prior to placement out-performing those who had non-immersive video, could be partially attributed to this teaching modality. Clinical placement evaluation represents a holistic assessment of practical, applied skills, problem-solving, and professional conduct in a simulated or real clinical environment, extending beyond rote memorization. The core advantage of immersive videos, as confirmed by perception data, is its ability to create a highly realistic and immersive environment. This immersive experience allows for more authentic practice and skill rehearsal, which appears to be directly transferable to clinical scenarios. As supported by the theory of multimedia cone of abstraction, virtual reality, or in this study immersive videos, is considered less abstract than video [18]. The less abstract nature of the immersive videos was postulated to aide with the familiarization of the procedures and the clinical reasoning behind them, as supported by our results. Students were not only able to grasp the execution of the procedures better, but given the realism of the videos, their learning of the underpinnings were more solid as a result.

Apart from being less abstract, superior transfer of procedural skills in students using immersive video could be explained by the theory of embodied cognition [19]. Unlike non-immersive video, which positions the student as a detached observer, the 360-degree immersive format allows for viewing the scenarios in a first-person perspective. This facilitates mental rehearsal as a clinician. By placing the student at the center of the clinical environment, immersive video bridges the gap between seeing a procedure and conceptually ‘doing’ it. This likely explains why the immersive group showed significantly higher competence in procedural tasks, such as oromotor examinations and swallow trials (*p* < 0.05), compared to the non-immersive group.

The results suggests that immersive technologies are particularly valuable for developing high-fidelity practical skills and clinical judgment in areas like dysphagia, where hands-on experience is paramount but often limited. It implies that VR’s strength lies in the application and synthesis of knowledge in a simulated clinical context. Since the videos used in this study covered the major settings in clinical practicum, participants in the immersive video group were able to have a more comprehensive and holistic understanding of the clinical environment prior to their placements. This pre-practicum familiarization could have facilitated better performance in clinical placements, from implementation of tasks, to interprofessional interaction and demonstration of clinical reasoning.

### Interpretation of perception differences and trade-offs

A critical observation from this study is the apparent paradox between the immersive group’s higher reported mean sense of presence and realism versus their lower mean overall satisfaction, ease of use, and recommendation likelihood. While immersive technology excels at delivering a heightened sense of presence and realism, its current implementation, encompassing hardware ergonomics, accessibility, and integration into learning workflows (particularly regarding note-taking), significantly detracts from the overall user experience. This leads to lower mean student satisfaction and recommendation rates when compared to the more straightforward non-immersive videos. The qualitative feedback directly explains this paradox by detailing specific pain points, such as headset weight, dizziness, difficulty taking notes, logistical issues with device sharing, and visual distortions. This indicates that the initial “novelty effect” of VR may be quickly overshadowed by practical usability issues, impacting its perceived value and adoption. The was concurrent with the finding that students in the non-immersive group spent more than three times as long viewing the materials, suggesting a significant difference in technological tolerability. Immersive videos or VR learning is likely to be more suitable for high-intensity, short-duration procedural training, its current lack of tolerability may hinder its use for conceptual review or long-form study.

This finding serves as a critical lesson for educational technology developers and implementers: cutting-edge technology, no matter how pedagogically promising, will face significant adoption challenges if it fails to prioritize user comfort, seamless accessibility, and integration into existing learning habits. The appeal of new technology must be balanced with practical usability to achieve sustained positive user experience and widespread acceptance.

According to the ARCS-V model proposed by Keller [20], when developing learning experiences, attention, relevance, confidence, satisfaction and volition need to be considered. The results from this study showed that attention, relevance and satisfaction did not seem to differ. For volition, the use of immersive videos seem to be limited by technological barriers which hinders the intrinsic and extrinsic volition in adopting this pedagogy. The lack of readily available instruments and the discomfort, along with the time required for adaptation, seem to lower the volition of students in adopting this technology in dysphagia education. Thus for curriculums which are considering this approach, the technological issues should be prioritized, perhaps through supplying more VR headsets and placing more emphasis on the technology during the course, to improve the adoption as supported by the components of the ARCS-V model.

### Implications for SLP dysphagia education

For dysphagia education, a carefully designed blended learning approach that strategically leverages the distinct strengths of both immersive and non-immersive video modalities appears to be the most promising pedagogical direction. Immersive videos could be reserved for critical, high-stakes clinical procedures that demand a strong sense of presence and procedural familiarity, thereby maximizing the development of applied skills. Conversely, non-immersive videos could function as accessible, easy to access and apply tools for conceptual understanding, repeated review of foundational material, and broad exposure to a wide array of clinical cases, ensuring flexibility and widespread access. This approach acknowledges that neither modality is a universal solution but that combining their strengths offers the most effective and practical solution for comprehensive dysphagia education.

### Limitations

This study is subject to several limitations. The participant profile (93.8% female) was heavily skewed towards female, and research has found that female students favour collaborative learning environments more [21]. This could be a potential confounding factor. Although male participants were split equally (*n* = 2 per group), future research with more balanced representation would help determine consistency across diverse student bodies. The specific sample size and the duration of the intervention may limit the generalizability of the findings. The particular VR hardware utilized (Meta Quest headset) may have influenced the reported usability challenges, and different platforms might yield varied results. The inherent subjectivity of self-reported perception data also warrants consideration. Furthermore, the presence of missing data (25% in clinical practicum performance and perception data) could potentially impact statistical power or the generalizability of the findings. Finally, the possibility of a “novelty effect” influencing the immersive group’s initial perceptions and performance cannot be entirely ruled out.

### Future research

Future studies should consider conducting longitudinal research to assess the long-term retention of knowledge and skill transfer from both immersive and non-immersive learning environments to actual clinical practice. Expanding the study to include other clinical aspects in SLP training would enhance the generalizability of the findings. Investigating the efficacy of different VR platforms and exploring the integration of interactive elements, such as in-VR note-taking features, could address some of the current usability challenges. Additionally, performing cost-effectiveness analyses of immersive versus non-immersive approaches would provide valuable information for educational institutions. A direct comparison of skill transfer to actual patient encounters would also represent a valuable future step in this line of inquiry.

## Conclusion

This study provides valuable insights into the comparative utility of immersive and non-immersive video learning in dysphagia education for SLP students. Immersive videos offer unique advantages in fostering a heightened sense of presence and demonstrate potential for enhancing clinical placement performance. However, these benefits are currently accompanied by significant usability and logistical challenges related to hardware ergonomics and accessibility. In contrast, non-immersive videos provide high accessibility and user satisfaction, leading to more sustained engagement and viewing time, albeit with a comparatively lower perceived sense of immersion.

The findings underscore the significant potential for a thoughtfully designed blended learning approach to optimize dysphagia education. Such an approach would strategically leverage the distinct strengths of both immersive and non-immersive modalities to create a more comprehensive and effective learning experience. Ultimately, the successful integration of novel technologies into the dynamic landscape of medical education necessitates a meticulous consideration of both pedagogical effectiveness and practical usability.

## Supplementary Information

Supplementary Material 1.

Supplementary Material 2.

## Data Availability

Data available upon reasonable request.

## Abbreviations

AR: Augmented Reality
ARCS-V: Attention, Relevance, Confidence, Satisfaction, Volition
OSCE: Objective Structured Clinical Examination
SD: Standard Deviation
SLP: Speech-Language Pathology
VR: Virtual Reality
