# Supplementary material for: The impact of immersive video learning on speech-language pathology students’ dysphagia education: a mixed-methods study
Source: BMC Med Educ. 2026 Jan 27;26:352. doi: 10.1186/s12909-026-08630-z (PMC12947463; doi:10.1186/s12909-026-08630-z)
Supplement: Supplementary file 1 — Supplementary Material 1. [file 12909_2026_8630_MOESM1_ESM.docx]

Supplementary material 1

Questionnaire to clinical supervisors on practicum performance

|  | Question | Response | | | | |
| --- | --- | --- | --- | --- | --- | --- |
| 1 | Years of experience as a CE for adult clinical placements (regardless of institution / setting) | (open-ended) | | | | |
| 2 | Nature of CURRENT placement setting | Nursing home | Daycare center | Community Rehabilitation Network center | | Others |
| 3 | No. of sessions completed to date | 1 | 2 | 3 | 4 | 5 |
| 4 | Name of student #1 | (open-ended) | | | | |
|  |  | Far below expectation | Below expectation | Average | Above expectation | Exceeding expectation |
| 5 | Assessment planning | 1 | 2 | 3 | 4 | 5 |
| 6 | Familiarity with placement setting / environment | 1 | 2 | 3 | 4 | 5 |
| 7 | Clinical procedures -Communication assessment | 1 | 2 | 3 | 4 | 5 |
| 8 | Clinical procedures – Oromotor assessment | 1 | 2 | 3 | 4 | 5 |
| 9 | Clinical procedures - Swallow trials | 1 | 2 | 3 | 4 | 5 |
| 10 | Handling of clinical material | 1 | 2 | 3 | 4 | 5 |
| 11 | Clinical reasoning | 1 | 2 | 3 | 4 | 5 |
| 12 | Interaction with patient / carer | 1 | 2 | 3 | 4 | 5 |
| 13 | Time management | 1 | 2 | 3 | 4 | 5 |
| 14 | Were there more student(s) in your group? | Yes (Q4 – Q14 repeated) | | | No (end of questionnaire) | |
