# Supplementary material for: The impact of immersive video learning on speech-language pathology students’ dysphagia education: a mixed-methods study
Source: BMC Med Educ. 2026 Jan 27;26:352. doi: 10.1186/s12909-026-08630-z (PMC12947463; doi:10.1186/s12909-026-08630-z)
Supplement: Supplementary file 2 — Supplementary Material 2. [file 12909_2026_8630_MOESM2_ESM.docx]

Supplementary material 2

Questionnaire on student perceptions and views

|  | **Question** | **Response** | | | | |
| --- | --- | --- | --- | --- | --- | --- |
| 1 | Your group number for the semester: | (open-ended) | | | | |
| 2 | You were provided with: | - immersive videos (Meta quest headset preloaded with videos) | | | | |
|  |  | - non-immersive videos (Link to session videos) | | | | |
| 3 | How many times did you view the video? | 0 | 1 | 2-3 | | >3 |
| 4 | How much time (roughly in terms of MINUTES) did you spend on viewing the videos throughout the whole learning period (Mar - Apr)? | (open-ended) | | | | |
|  | **Engagement** |  |  |  |  |  |
| 5 | How actively involved did you feel while using the learning material? | 1 (Not involved at all) | 2 | 3 | 4 | 5 (Very involved) |
| 6 | Did you find the learning material (video) engaging? | 1 (Not engaging) | 2 | 3 | 4 | 5 (Extremely engaging) |
| 7 | How well did the material hold your attention throughout the viewing? | 1 (Not at all) | 2 | 3 | 4 | 5 (Completely) |
|  | **Immersion** |  |  |  |  |  |
| 8 | To what extent did you feel "present" in the environment depicted in the video? | 1 (Not present) | 2 | 3 | 4 | 5 (Fully immersed) |
| 9 | How realistic did the scenarios feel? | 1 (Not realistic at all) | 2 | 3 | 4 | 5 (Extremely realistic) |
| 10 | Did the videos help with familiarising you with the clinical setting? | 1 (Not at all) | 2 | 3 | 4 | 5 (Completely) |
| 11 | Did the videos help with familiarising you with the clinical procedures? | 1 (Not at all) | 2 | 3 | 4 | 5 (Completely) |
| 12 | Did the videos help with your understanding of clinical reasoning? | 1 (Not at all) | 2 | 3 | 4 | 5 (Completely) |
|  | **Satisfaction** |  |  |  |  |  |
| 13 | Did the learning material meet your expectations? | 1 (Not at all) | 2 | 3 | 4 | 5 (Exceeded expectations) |
| 14 | How likely are you to recommend this learning method to other students? | 1 (Not likely) | 2 | 3 | 4 | 5 (Very likely) |
|  | Usability |  |  |  |  |  |
| 15 | How easy was it to navigate and use the learning material? | 1 (Very difficult) | 2 | 3 | 4 | 5 (Very easy) |
| 16 | Did you experience any technical issues while using the material? | Yes, (open-ended) | | | | No |
| 17 | Were the instructions for using the material clear and helpful? | 1 (Not clear) | 2 | 3 | 4 | 5 (Extremely clear) |
|  | **Accessibility** |  |  |  |  |  |
| 18 | Were you able to access the learning material without any difficulties? | 1 (Not accessible) | 2 | 3 | 4 | 5 (Completely accessible) |
| 19 | Did you require additional assistance (e.g. from classmates, friends, web search) to use the learning material? | Yes, (open-ended) | | | | No |
| 20 | How accessible do you think this method would be for students with different needs (e.g., physical, visual, or sensory challenges)? (1= Not accessible, 5= Extremely accessible) | 1 (Not accessible) | 2 | 3 | 4 | 5 (Completely accessible) |
|  | **Perceived barriers** |  |  |  |  |  |
| 21 | What challenges did you face while using the learning material? | (open-ended) | | | | |
| 22 | Did you find the technology overwhelming or intimidating? | 1 (Not at all) | 2 | 3 | 4 | 5 (Very much so) |
| 23 | How much time did it take to get comfortable with the learning material? | 1 (No time at all) | 2 | 3 | 4 | 5 (Significant amount of time) |
| 24 | Were there any aspects of the learning method that  hindered your learning? | (open-ended) | | | | |
| 25 | What improvements would you suggest to enhance the learning experience? | (open-ended) | | | | |
|  | **Overall satisfaction** |  |  |  |  |  |
| 26 | How satisfied were you with the overall quality of the learning experience? | 1 (Not satisfied) | 2 | 3 | 4 | 5 (Extremely satisfied) |
